# Food quality, security, and thermal refuge influence the use of microsites and patches by pygmy rabbits (*Brachylagus idahoensis*) across landscapes and seasons

**DOI:** 10.1002/ece3.8892

**Published:** 2022-05-13

**Authors:** Peter J. Olsoy, Charlotte R. Milling, Jordan D. Nobler, Meghan J. Camp, Lisa A. Shipley, Jennifer S. Forbey, Janet L. Rachlow, Daniel H. Thornton

**Affiliations:** ^1^ 6760 School of the Environment Washington State University Pullman Washington USA; ^2^ 1791 Department of Biological Sciences Boise State University Boise Idaho USA; ^3^ School of Environment and Natural Resources Ohio State University Columbus Ohio USA; ^4^ Department of Fish and Wildlife Sciences University of Idaho Moscow Idaho USA

**Keywords:** food quality, functional properties, multi‐scale habitat use, predation risk, security cover, thermal refuge

## Abstract

How intensely animals use habitat features depends on their functional properties (i.e., how the feature influences fitness) and the spatial and temporal scale considered. For herbivores, habitat use is expected to reflect the competing risks of starvation, predation, and thermal stress, but the relative influence of each functional property is expected to vary in space and time. We examined how a dietary and habitat specialist, the pygmy rabbit (*Brachylagus idahoensis*), used these functional properties of its sagebrush habitat—food quality, security, and thermal refuge—at two hierarchical spatial scales (microsite and patch) across two seasons (winter and summer). At the microsite and patch scales, we determined which plant functional traits predicted the number of bites (i.e., foraging) by pygmy rabbits and the number of their fecal pellets (i.e., general habitat use). Pygmy rabbits used microsites and patches more intensely that had higher crude protein and aerial concealment cover and were closer to burrows. Food quality was more influential when rabbits used microsites within patches. Security was more influential in winter than summer, and more at Cedar Gulch than Camas. However, the influence of functional properties depended on phytochemical and structural properties of sagebrush and was not spatiotemporally consistent. These results show function‐dependent habitat use that varied according to specific activities by a central‐place browsing herbivore. Making spatially explicit predictions of the relative value of habitat features that influence different types of habitat use (i.e., foraging, hiding, and thermoregulating) will improve how we predict patterns of habitat use by herbivores and how we monitor and manage functional traits within habitats for wildlife.

## Graphical abstract

We examined how a dietary and habitat specialist, the pygmy rabbit (*Brachylagus idahoensis*), responded to functional properties of its habitat—food quality, security, and thermal refuge—at two hierarchical spatial scales (microsite and patch) across two seasons (winter and summer) at two study sites. In general, pygmy rabbits used microsites and patches with higher crude protein and aerial concealment cover closer to burrows. These results show function‐dependent habitat selection that varies according to specific functional uses by a central‐place browsing herbivore.

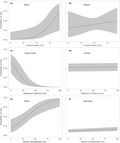

## 1 INTRODUCTION

Natural selection favors animals that select habitats that advance lifetime fitness. However, because different habitat features often provide different fitness benefits (i.e., functional properties) to animals, individuals must often trade‐off required resources, such as choosing between abundant nutritious forage and high predation risk or poor forage and low predation risk (McArthur et al., 2014). When animals decide which habitats to use, the most influential habitat features might change depending on the functional use of the resource, such as foraging, hiding, or mating (Godvik et al., 2009). For example, habitat use is function‐dependent in greater sage‐grouse (*Centrocercus urophasianus*), which selected habitats with dwarf sagebrush (e.g., *Artemisia nova*) in winter for food (Frye et al., 2013), big sagebrush (*Artemisia tridentata*) for cover during nesting, and open leks for mating in early spring (Connelly et al., 2000). Habitat use by animals can also depend on the specific local properties (i.e., context) of the site or season (Perea et al., 2013). For small herbivores, seasonal growth and senescence of plant biomass inherently alter patterns of food availability and nutritional quality (Gregg et al., 2008), security, and thermal refuge across the landscape (Milling, Rachlow, Olsoy, et al., 2018).

The value of different habitat features also depends on the spatiotemporal scales at which animals use them (Apps et al., 2001; Ciarniello et al., 2007; Pinaud & Weimerskirch, 2005; Rettie & Messier, 2000). Fitness outcomes are linked to resource requirements that are not constant across space and time (Bailey et al., 1996; Senft et al., 1987). Therefore, herbivores must balance the varying and competing risks of starvation, predation, and thermal stress (Hebblewhite & Merrill, 2009; Kotliar & Wiens, 1990) at different spatial and temporal scales. At the broadest scale, a species’ range might be determined by physiological limitations or access to all key resources across the time span of the species or population (Senft et al., 1987). However, at mesoscales, such as the animal's home range (used across its lifespan) or landscape, and micro‐scales, such as food patches or hibernacula used over minutes to months, the relative influence of food, security, and thermal refuge might shift, depending on the system and species (Hebblewhite & Merrill, 2009; Rettie & Messier, 2000).

Biologists often choose to use habitat metrics when modeling habitat use and selection that are quick and easy to measure (e.g., plant height and species composition) rather than directly measuring the functional properties that these habitat features might provide. In addition, studies are rarely designed to capture how animals use specific habitat resources that are critical for multiple, yet distinct, functional uses by the animal across multiple scales (Frye et al., 2013; Hebblewhite & Merrill, 2009; Wiens, 1989). Evaluating functional uses of habitat features by animals at multiple scales provides the mechanistic underpinning of habitat use necessary to quantify and manage habitats in ways functionally meaningful to animals.

Our goal was to measure habitat use at two hierarchical spatial scales and two seasons, where both resource availability and herbivore life requisites differed. We evaluated the use of habitat features by a small (~450 g) dietary and habitat specialist, the pygmy rabbit (*Brachylagus idahoensis*). Pygmy rabbits are endemic to the Western USA (Smith et al., 2019) and are restricted to large, relatively continuous patches of sagebrush growing on deep, friable soils (Weiss & Verts, 1984). Pygmy rabbits eat 2–8 times more sagebrush in the winter than in summer (Thines et al., 2004), and the diet composition of sagebrush can vary 2‐ to 4‐fold among sites within a season based on available forages (Crowell et al., 2018). Sagebrush contains relatively high digestible protein and toxic plant secondary metabolites (PSMs) year‐round, but these concentrations vary by species, habitat, and season (Robb, 2020). Pygmy rabbits are subject to heavy predation from aerial and terrestrial predators (Green & Flinders, 1980; Price et al., 2010; Wilde, 1978) and inhabit a thermally variable environment (Milling et al., 2017). Landscapes used by pygmy rabbits also contain areas of deep soil that provide a resource used by pygmy rabbits to dig burrows that serve as refuges from predation (Price et al., 2010; Sanchez & Rachlow, 2008) and daily and annual temperature extremes (Milling et al., 2017; Rachlow et al., 2005). Pygmy rabbits are active year‐round and rely on sagebrush plants for food, aerial and terrestrial concealment (Camp et al., 2012), and thermal buffering (Milling et al., 2017). Previous studies in experimental arenas quantified the marginal value of the relative risks of nutrients and toxins in food, concealment, distance to a burrow refuge, and temperature when choosing food patches (Camp et al., 2015, 2017; Milling, Rachlow, Chappell, et al., 2018; Nobler et al., 2019). How pygmy rabbits evaluate these risks in natural habitats requires measuring the functional properties of habitat features (contributions to food quality, security, and thermal refuge) between spatial scales and seasons across landscapes.

We measured the functional properties of the microsite centered on an individual sagebrush shrub and the surrounding microclimate (microsite scale) and sagebrush patches (i.e., a group of sagebrush plants and surrounding microclimate) to pygmy rabbits within two landscapes (Camas and Cedar Gulch) in Idaho, USA. Specifically, we measured values of food quality (nutrients and toxic PSMs in sagebrush), security (aerial and terrestrial concealment and distance to burrow refuge), and thermal refuge (operative temperature variables and distance to a burrow). Simultaneously, we measured two distinct functional uses of microsites (i.e., the plant and 0.5‐m area around each plant) and patches by pygmy rabbits, which included (1) counts of bite marks on sagebrush that represented active foraging and (2) counts of fecal pellets that represented general habitat use as pellets are deposited during various behaviors including moving, resting, and foraging. Because the phytochemical, structural, and thermal conditions (i.e., context) differed between the two study sites (Camas and Cedar Gulch), we first hypothesized (H1) that food would be the most influential habitat feature of microsites and patches used at Camas, which has lower nutrients and higher PSMs (Olsoy et al., 2020), and security and thermal cover to be more influential at Cedar Gulch, which has a greater range in seasonal temperatures (Milling, Rachlow, Chappell, et al., 2018; Milling et al., 2017; Milling, Rachlow, Olsoy, et al., 2018) and shorter and more sparsely distributed sagebrush (Olsoy et al., 2018). Second, we hypothesized that the influence of different functional properties (nutrients, toxins, security, and thermal refuge) would vary based on how the rabbits used the microsite or patch (i.e., function‐dependence). We expected (H2) food to be more influential when rabbits used microsites and patches for foraging (bites) than for general use (fecal pellets). Third, we hypothesized that rabbits would use habitat in a hierarchical manner whereby the influence of food and security would differ across spatial scales. Because patches are used at longer time scales than microsites and contain a mixture of sagebrush and other plants, more burrows, and a variety of other habitat features, we expected (H3) security to more strongly influence the use of patches (Bailey et al., 1996; Johnson et al., 2002; Senft et al., 1987), and food to determine the plant on which to feed within the patch. Fourth, because of seasonal differences in temperature and availability of alternative less‐toxic foods like grasses and forbs (Thines et al., 2004), we hypothesized (H4) that the relative value of food, security, and the thermal refuge would vary between winter and summer (i.e., seasonal context). We expected the quality of sagebrush (food) to influence habitat use more strongly during winter when it forms the bulk of the pygmy rabbit's diet than in summer when rabbits eat a greater variety of forages. Furthermore, because pygmy rabbits are more sensitive to extremely high temperatures than low temperatures in our study sites (Milling et al., 2017), we expected thermal refuge to be more influential during summer than winter.

## 2 METHODS

### 2.1 Study sites

**FIGURE 1:**
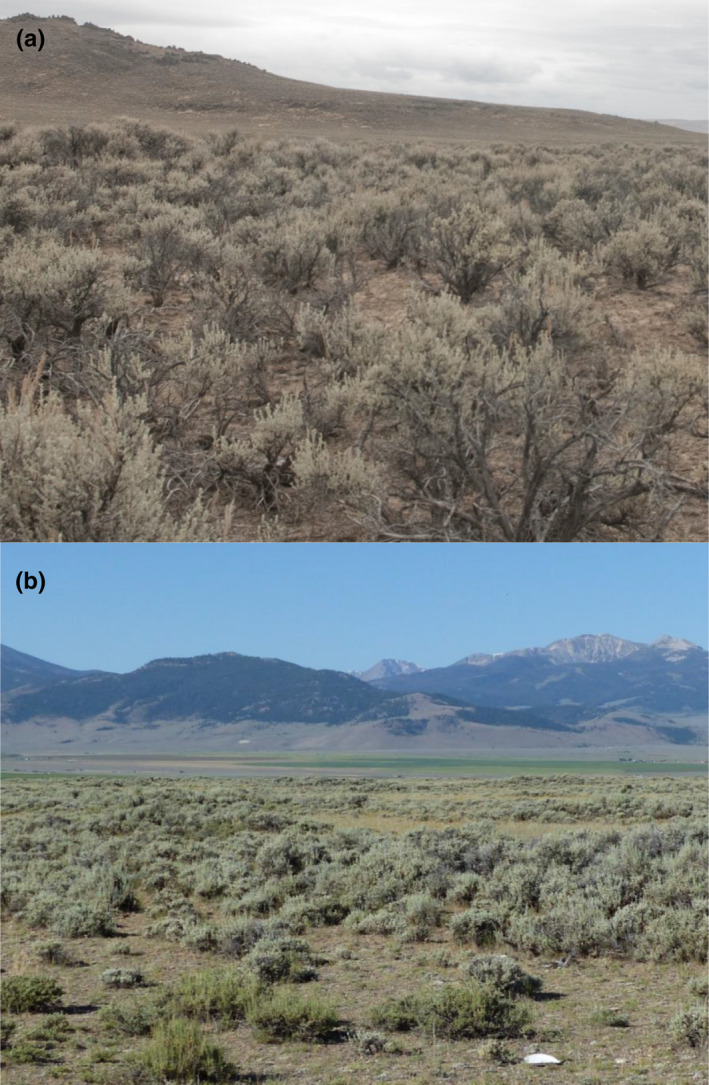
Photographs of vegetation communities at the (a) Camas and (b) Cedar Gulch study sites in Idaho, USA

**FIGURE 2:**
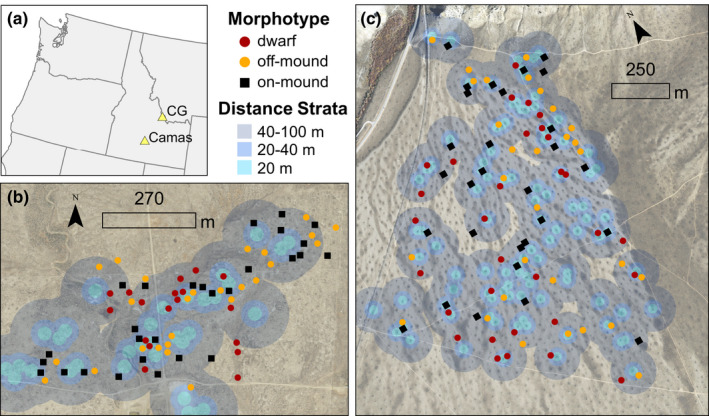
Sagebrush (*Artemisia* spp.) morphotypes (i.e., patch types) at two study sites in Idaho, USA (a): Camas (b) and Cedar Gulch (c). Patches were selected in a stratified random design based on the distance from active pygmy rabbit (*Brachylagus idahoensis*) burrows (i.e., distance strata)

We conducted research at two sites in Idaho, USA (Figures 1 and 2). The Camas site (lat 43°14′28″ N, long 114°19′04″ W, elevation 1465–1480 m) is an area of ~55 ha located in southcentral Idaho. We completed fieldwork at the Camas site in January 2014 (winter) and June 2014 (summer). Average temperatures (2000–2022) in January were −1.0°C, 20.4°C in June, and the site received 27.2 cm precipitation annually. The year of our study at Camas (2014) averaged 1.1°C hotter annually but with cooler average January and June temperatures (−1.4 and 18.2°C, respectively) and 10.8 cm greater in precipitation (National Weather Service, 2022) than the 22‐year average. The Cedar Gulch site (lat 44°41′57″ N, long 113°17′12″ W, elevation 1885–1925 m) is an area of ~155 ha in eastern Idaho, along the Montana border. We completed fieldwork at the Cedar Gulch site in January 2015 (winter) and June 2015 (summer). Average temperatures (2000–2022) in January were −5.4°C, 16.6°C in June, and the site received on average 28.5 cm precipitation annually. The year of our study at Cedar Gulch (2015) averaged 2.1°C hotter annually with average January temperatures of −4.1°C, June temperatures of 20.4°C, and 4.0 cm greater precipitation than the 22‐year average (National Weather Service, 2022). Wyoming big sagebrush (*A*. *tridentata* subsp. *wyomingensis*) dominated both study sites on earthen mima mounds (on‐mound patches representing deeper soils) and off‐mound patches. Short‐statured “dwarf” patches of sagebrush also were common in the matrix between mounds. At Camas, the dwarf patches were composed of another species of sagebrush (low sagebrush, *Artemisia arbuscula*), whereas at Cedar Gulch, the dwarf patches were primarily low‐growing Wyoming big sagebrush mixed with black sagebrush (*A*.* nova*). The plants within on‐mound, off‐mound, and dwarf patches differed in dietary (Olsoy et al., 2020) and structural traits (Olsoy et al., 2018). Other lagomorphs at both study sites included mountain cottontails (*Sylvilagus nuttallii*) and black‐tailed jackrabbits (*Lepus californicus*). Both sites had diverse avian and mammalian predators (Estes‐Zumpf & Rachlow, 2009).

### 2.2 Data collection

We collected field data in a hierarchical sample design focused on areas determined as a high activity to maximize our chances of detecting pygmy rabbit use. We conducted complete burrow surveys using belt transects at each study site to determine the location of mounds with active burrows based on whether burrow entrances were open or collapsed and signs of use (e.g., fresh digging, recent vs. old fecal pellets; Sanchez et al., 2009). From the locations of currently active burrows, we stratified each study site into three groups based on distance from potential pygmy rabbit activity: high (0–20 m from an active burrow), medium (20–40 m), and low activity (40–100 m) (Figure 2). Then, to select our sample patches within each distance strata, we generated random points ≥20 m apart. In the field, we selected the first 10 random points that correctly corresponded to each of the three patch types within each distance stratum (total *n* ~ 90) (Figure 2). Each patch type (on‐mound, off‐mound, or dwarf) contained the same sagebrush morphotype within a 5‐m radius area. In some cases, too few patches were located within the higher active stratum, so fewer patches per stratum were sampled (*n* = 70 patches at Camas, *n* = 88 patches at Cedar Gulch). At Camas, a few dwarf patches had to be selected outside of the activity strata because of the extreme separation between dwarf patches and most active mounds (Figure 2). Within each patch, we randomly selected three focal plants (within patches with a single sagebrush species) or six focal plants (within multi‐species patches) as the center for microsite sampling (*n* = 209 plants at Camas and *n* = 290 plants at Cedar Gulch).

We sampled each focal plant and the microsite environment around the plant (0.5‐m radius) for use by pygmy rabbits. Microsite use was measured by counting the number of bite marks on the focal plant created during browsing (active foraging) and counting the number of fecal pellets within a 0.5‐m radius of focal plants (general use of the microsite). Pygmy rabbit bite marks can be distinguished by stems clipped at a 45° angle with stem diameter ≤2 mm, whereas mountain cottontails discard leaves and forage on stems. We only counted bites with green stems, indicating fresh bites. Pygmy rabbit pellets are distinguishable from mountain cottontails and jackrabbits based on their small size (~0.5 cm diameter). The lack of larger lagomorph pellets was used to confirm the attribution of small diameter browsing to pygmy rabbits. To assess use at the patch scale, we determined if the patch was used (1) or unused (0) based on bites and pellets from all focal plants within the patch. Additionally, if no focal plants were browsed and no pellets were found near the focal plants, then patch‐scale use was further assessed with a brief (10 person‐min) search for additional pellets within the patch.

We measured functional properties associated with forage quality, security cover, and thermal refuge at each microsite. We measured two components of food quality, one representing nutrient content and the other the concentration of a dominant PSM (i.e., monoterpenes), both of which influenced diet and patch choices in controlled experiments with captive pygmy rabbits (Camp et al., 2015). Crude protein represented the forage quality of the plant, whereas total monoterpenes represented toxins that might decrease herbivory (Crowell et al., 2018; Nobler et al., 2019). We collected leaf samples for lab analysis of crude protein and monoterpenes. Briefly, we clipped leaf samples to collect about 2 g of fresh mass from each plant and stored them on the ice during transport to the lab. We stored leaf samples at −20°C, ground them in liquid nitrogen, and separated subsamples for crude protein and monoterpene analysis. For crude protein, the ground subsample was dried and sent to Dairy One Forage (Ithaca, NY) for nitrogen analysis using the Dumas combustion method (Etheridge et al., 1998). We converted total nitrogen to crude protein by multiplying by 6.25 because the average nitrogen content of amino acids is 16% (Robbins, 1983). For monoterpene analysis, we used headspace gas chromatography (Agilent 7694 Headspace Sampler, Agilent 6890 Series Gas Chromatograph) to quantify all individual monoterpenes detected before the retention time of 24 min (Nobler, 2016). We quantified total monoterpenes as the sum of the area under the curve (AUC) for each chemical peak detected divided by the dry weight of the sample (AUC/mg DW).

We measured three components of security cover—aerial concealment, terrestrial concealment, and distance to an active burrow—all of which were associated with patch selection in controlled experiments with captive pygmy rabbits (Camp et al., 2017; Crowell et al., 2016; Milling, Rachlow, Chappell, et al., 2018). Aerial and terrestrial concealment and distance to burrow represent security because pygmy rabbits retreat to burrows to escape predation (Camp et al., 2012) and use sagebrush cover to conceal themselves from possible predators when outside the burrow. At each focal plant, we estimated aerial concealment by placing four 15 × 15‐cm cover boards divided into 25 cells under each plant and taking a digital photograph directly over the plant from a height of 1.5 m. We recorded the number of cells that were ≥50% obscured by vegetation as an index of aerial concealment (Camp et al., 2013; Nobler, 2016). For terrestrial concealment (i.e., horizontal concealment from the perspective of a terrestrial predator), we placed a 15 × 15 × 15‐cm profile cube similar to the size of a pygmy rabbit within the habitat at the base of each focal plant. Each side of the cube was viewed from 4 m at a height of 1 m above the ground in all four cardinal directions and counted cells that were ≥50% concealed to attain an estimate of percent terrestrial concealment (Camp et al., 2012; Milling et al., 2017; Olsoy et al., 2015). To determine the distance to an active burrow, we used a survey‐grade GPS receiver (Topcon, Livermore, California) to measure the location of each focal plant from the active burrows we had located previously.

We measured two functional properties of thermal refuge of microsites and patches, distance to burrow and operative temperature. Burrows are used by pygmy rabbits to buffer thermal extremes, where they were up to 15°C warmer in winter and 20°C cooler in summer in our study area (Milling, Rachlow, Chappell, et al., 2018). We measured operative temperature using biophysical models at the base of each plant with a matte black hollow sensor designed to mimic how an animal lacking metabolic heating and cooling experiences the thermal environment (Milling, Rachlow, Chappell, et al., 2018). The sensors were left within a microsite at a focal plant in each patch for 2 weeks and then rotated through the patch until each focal plant had been sampled during both summer and winter. We measured the average daily maximum and minimum temperatures and average diurnal temperature range (DTR) for each focal plant during both seasons. Because temperatures varied across sampling periods in each season, we standardized temperature values by the mean value for the sampling period, treating the sampling period as a blocking variable (see Milling, Rachlow, Chappell, et al., 2018).

At the patch scale, we averaged the microsite food quality measurements and modeled patch‐scale security and thermal refuge. We assumed that for food quality, the focal plants were an unbiased sample that represented the crude protein and total monoterpenes of the entire patch. For security, we used structural data from unoccupied aerial systems (UAS) to estimate aerial concealment for the entire patch (see Olsoy et al., 2018 for full details on UAS methods). We calculated the patch‐scale distance to the burrow as the Euclidean distance to the nearest active burrow (averaged over the 5‐m radius patch). For patch‐scale thermal refuge, we modeled operative temperature across the landscape as described in Milling, Rachlow, Olsoy, et al. (2018) with UAS‐derived aerial concealment and plant volume (data from Olsoy et al., 2018), and a classified map of sagebrush morphotypes (data from Olsoy et al., 2020) as input variables into the thermal models.

### 2.3 Statistical analyses

At the microsite and patch scales, we examined the functional food, security, and thermal variables that influenced foraging or general use by pygmy rabbits. Each model we tested represented a hypothesis about how pygmy rabbits chose microsites and patches based on their functional properties and included combinations of each variable (Table 1). We modeled the number of pygmy rabbit bites and fecal pellets at the microsite scale (response variables) with negative binomial generalized linear mixed models (GLMM) with a log‐link function in the “lme4” R package (Bates et al., 2015). We used a negative binomial model because of the high proportion of zeros in our dataset at the microsite scale. Because the microsite samples were collected hierarchically with 3–6 plants within each patch, the patch was included as a random intercept in all models. We generated a separate set of models for each site (Camas and Cedar Gulch) and season (winter and summer). We scaled monoterpenes by subtracting the mean and dividing by the standard deviation because values ranged from 44–2182 AUC/mg DW, where variables with high values can lead to model convergence issues. We checked predictor variables for multicollinearity, and when variables were correlated (*r* > .7), we retained the most biologically relevant variables (Appendix S1, Tables S1–S8). Aerial and terrestrial concealment were correlated, so we kept aerial concealment because it varied among sagebrush morphotypes and could be mapped across the landscape using remotely sensed imagery (Olsoy et al., 2015, 2018). Minimum and maximum temperature were correlated with each other and with DTR, so we chose to use only DTR in our models because we expected that pygmy rabbits would avoid greater temperature ranges during both winter and summer (Milling et al., 2017). We ranked models with Akaike's Information Criterion corrected for small sample sizes (AIC_c_), and we summed the AIC_c_ weights for each hypothesis category (food, security, and thermal) to rank support for each hypothesis on habitat use by pygmy rabbits. We calculated the marginal‐R^2^ for GLMMs (Johnson, 2014) for each top model with the “MuMIn” R package (Barton, 2019) and further evaluated fit with simulated residual tests in the “DHARMa” R package (Hartig, 2022) to assess the goodness of fit. Finally, we performed model averaging for all models ≤4 ΔAIC_c_ of the top model and standardized estimates based on partial standard deviations to account for multicollinearity (Cade, 2015).

**TABLE 1:** Model hypotheses and the functional properties contained within each model for predicting habitat use by pygmy rabbits (*Brachylagus idahoensis*) in Idaho

Model name	Variables	Hypotheses weighted
GLOBAL	CP + MT + AC + D2B + DTR	Food, security, thermal
FOOD + SECURITY	CP + MT + AC + D2B	Food, security
FOOD + THERMAL	CP + MT + D2B + DTR	Food, thermal
SECURITY + THERMAL	AC + D2B + DTR	Security, thermal
FOOD	CP + MT	Food
SECURITY	AC + D2B	Security
THERMAL	D2B + DTR	Thermal
CP	CP	Food
MT	MT	Food
AC	AC	Security
D2B	D2B	Security, thermal
DTR	DTR	Thermal
NULL	–	–

Note

AC, aerial concealment (%); CP, crude protein (%); D2B, distance to burrow (m); DTR, mean diurnal temperature range (°C); MT, total monoterpenes (scaled).

We modeled patch‐scale use with logistic regression. We ranked the patch‐scale models with AIC_c_ and summed AIC_c_ weights for each hypothesis as evidence of relative influence. We estimated the goodness of fit for the top model with McFadden's pseudo‐*R* ^2^ (McFadden, 1974), the AUC of the receiver operating characteristic curve (AUC_ROC_), and simulated residuals with the DHARMa R package (Hartig, 2022). We performed model averaging for all models ≤4 ΔAIC_c_ of the top model and standardized estimates based on partial standard deviations.

## 3 RESULTS

We documented low to moderate use by pygmy rabbits at both the microsite and patch scales. Only 4%–17% of individual sagebrush plants we sampled were browsed by pygmy rabbits (Table 2), and 4%–39% contained pellets within a 0.5‐m radius of the plant. Microsite‐scale negative binomial models explained a relatively small portion of the overall variation in use (*R* ^2^ = .01–.33). For patches, browsing was documented in 9%–22% of patches, and 9%–60% contained pellets somewhere within the patch. Patches within Cedar Gulch were more frequently used than patches within Camas during both seasons but use at Camas increased in winter (Table 2). Patch‐scale top models also varied substantially between study sites (Table 2). Logistic regression model fit was relatively low with an *R* ^2^ of .08–.59, but an AUC_ROC_ of .72–.94 indicated moderate accuracy in the ability of our model to distinguish used from unused patches (Table 2). Simulated residuals were normal for all models except microsite‐scale bites at Camas in summer, which showed signs of uniformity (Kolmogorov–Smirnov test) and dispersion (DHARMa nonparametric dispersion test), likely because 96% of plants had zero bite marks (Table 2).

**TABLE 2:** Top negative binomial generalized linear mixed models for microsite use (i.e., number of bites or number of pellets) and logistic regression models for patch‐scale use (i.e., the presence of pellets in the patch or bites on plants within the patch) by pygmy rabbits (*Brachylagus idahoensis*) at the Camas and Cedar Gulch study sites in Idaho, USA, during winter and summer

Scale	Use	Season	Sites	Percent zeros	AIC_c_ Weights			*R* ^2^	AUC_ROC_ ^c^
					Food	Security	Thermal		
Microsite	Bites	Winter	Camas	83	**0.433**	0.351	**0.510**	.030^a^	–
			Cedar Gulch	90	0.159	**0.825**	0.666	.327^a^	–
		Summer	Camas	96	**0.810**	0.137	0.150	.044^a^	–
			Cedar Gulch	93	**0.930**	0.391	**0.800**	.008^a^	–
	Pellets	Winter	Camas	82	**0.999**	0.053	0.083	.144^a^	–
			Cedar Gulch	75	0.221	0.474	**0.908**	.187^a^	–
		Summer	Camas	96	**0.385**	**0.279**	**0.295**	–^a^	–
			Cedar Gulch	61	0.507	**1.000**	0.508	.272^a^	–
Patch	Bites	Winter	Camas	78	0.308	**0.592**	**0.484**	.219^b^	0.848
			Cedar Gulch	82	0.571	**0.984**	0.263	.585^b^	0.935
		Summer	Camas	91	**0.507**	0.290	0.263	–	–
			Cedar Gulch	85	**0.831**	0.379	0.225	.104^b^	0.715
	Pellets	Winter	Camas	65	0.156	**0.674**	**0.674**	.098^b^	0.816
			Cedar Gulch	56	0.797	**0.999**	0.376	.395^b^	0.722
		Summer	Camas	91	**0.488**	**0.533**	**0.593**	.075^b^	0.865
			Cedar Gulch	40	0.053	**1.000**	0.284	.173^b^	0.693

Note

The sum of the AIC_c_ weights for each habitat selection hypothesis (food, security cover, and thermal refuge) are provided and the hypothesis with the most weight is bolded (if two or more hypotheses had summed weights within 0.15 they are all bolded). Tables with all the models tested are in Appendix S1, Tables S9–S28.

^a^ *R* ^2 ^M = marginal *R* ^2^, an estimate of the goodness of fit for the top model.

^b^ *R* ^2^_McF_ = McFadden's pseudo‐*R* ^2^ representing the relative goodness of fit of the top model.

^c^ AUC_ROC_ = area under the curve of the receiver operating characteristic curve for the top model.

**FIGURE 3:**
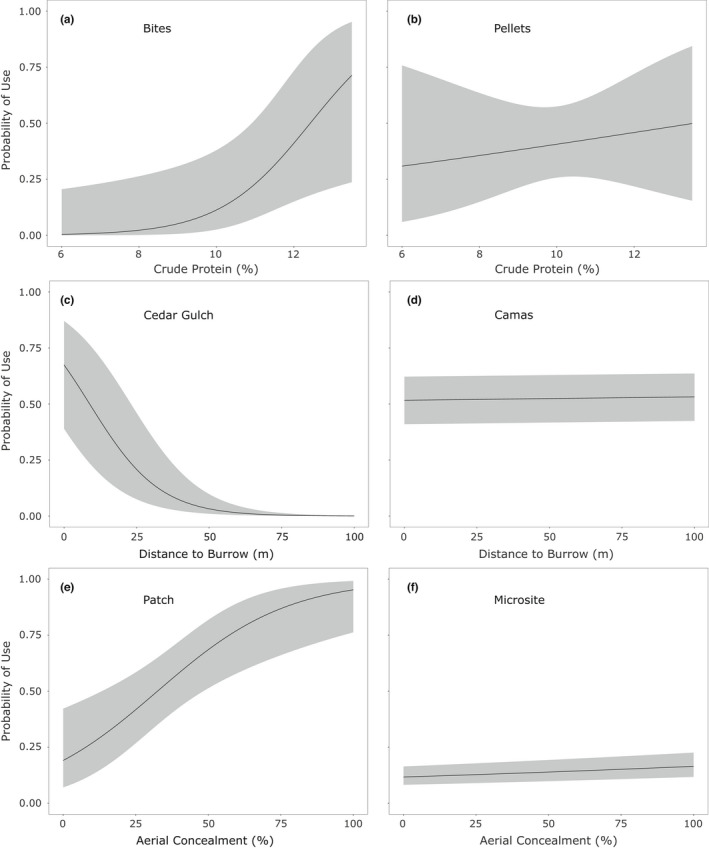
Response curves with 95% confidence intervals demonstrating the most extreme functional properties affecting the probability of use by pygmy rabbits (*Brachylagus idahoensis*) observed in our study in Idaho, including the influence of (a, b) crude protein on the use of patches for foraging (bites) versus general use (fecal pellets) during winter at the Camas site; (c, d) distance to burrow on the selection of microsites for general use at Cedar Gulch and Camas sites during winter; and (e, f) aerial concealment on use of both patches and microsites for general use at Cedar Gulch during winter

In assessing our hypotheses, there was equivocal support for a greater influence of food traits at Camas than at Cedar Gulch (H1) (Figure 3). Food was the most highly supported model (based on AIC weights) at Camas in 4 of the 8 model comparisons and competitive in 2 additional comparisons. In contrast, food appeared as the most highly supported model in only 2 of 8 comparisons for Cedar Gulch (Table 2). However, based on model‐averaged parameter estimates, strongly influential food variables (with confidence intervals around β estimates that did not overlap 0) were included equally for models at Cedar Gulch and Camas (3 of 8 vs. 3 of 8; Table 3). There was more consistent support for a greater influence of security at Cedar Gulch than at Camas (H1), with security being the most highly supported model in 5 of 8 comparisons for Cedar Gulch and 1 of 8 for Camas (but was competitive for 3 comparisons). Based on model‐averaged parameter estimates, security variables were highly influential in 6 of 8 comparisons at Cedar Gulch and only 3 at Camas. We found no support for a greater influence of thermal refuge in Cedar Gulch than at Camas (H1), with thermal refuge as the most highly supported model in only 1 of 8 Cedar Gulch comparisons, and 3 of 8 Camas comparisons (Table 2). Model‐averaged estimates of thermal variables showed highly influential thermal variables in 5 of 8 Cedar Gulch comparisons and only 3 of 8 Camas comparisons, but the specific individual variable that was influential was always distance to burrow (a variable that is also related to security).

**TABLE 3:** Model‐averaged parameter estimates with one standard error in parentheses for negative binomial generalized linear mixed models for microsite use (i.e., number of bites or number of pellets) and logistic regression models for patch‐scale use (i.e., the presence of pellets in the patch or bites on plants within the patch) by pygmy rabbits (*Brachylagus idahoensis*) at the Camas and Cedar Gulch study sites in Idaho, USA, during winter and summer

Scale	Use	Season	Site	CP	MT	AC	D2B	DTR
Microsite	Bites	Winter	Camas	0.59 (0.57)	0.02 (0.25)	0.29 (0.42)	−0.54 (0.82)	−0.38 (0.45)
			Cedar Gulch	0.09 (0.26)	0.13 (0.33)	0.18 (0.34)	−**4.59 (2.35)**	0.18 (0.42)
		Summer	Camas	0.65 (0.85)	1.07 (0.98)	−0.04 (0.16)	0.25 (0.70)	0.10 (0.30)
			Cedar Gulch	**1.19 (0.39)**	−0.50 (0.48)	0.28 (0.48)	−0.79 (0.49)	−0.53 (0.39)
	Pellets	Winter	Camas	**1.56 (0.39)**	0.00 (0.16)	−0.04 (0.18)	0.02 (0.22)	0.20 (0.33)
			Cedar Gulch	0.15 (0.24)	0.00 (0.12)	0.12 (0.20)	−**2.00 (0.60)**	−0.60 (0.31)
		Summer	Camas	−0.13 (0.40)	0.36 (0.63)	−0.04 (0.23)	−0.20 (0.58)	−0.07 (0.26)
			Cedar Gulch	0.34 (0.26)	0.06 (0.09)	**1.14 (0.16)**	−**0.90 (0.20)**	0.17 (0.18)
Patch	Bites	Winter	Camas	0.89 (0.56)	−0.01 (0.59)	0.65 (0.58)	−**1.04 (0.51)**	−0.57 (0.74)
			Cedar Gulch	−0.90 (0.52)	0.93 (0.72)	**1.90 (0.78)**	−**3.01 (0.99)**	0.04 (0.48)
		Summer	Camas	**0.90 (0.47)**	−0.46 (0.46)	−0.71 (0.50)	0.27 (0.47)	0.53 (0.55)
			Cedar Gulch	**1.01 (0.42)**	0.37 (0.38)	0.55 (0.42)	−0.61 (0.42)	−0.07 (0.40)
	Pellets	Winter	Camas	0.30 (0.38)	−0.20 (0.39)	−0.15 (0.35)	−**0.88 (0.40)**	0.12 (0.36)
			Cedar Gulch	−0.50 (0.31)	**0.73 (0.34)**	**1.26 (0.37)**	−**0.81 (0.37)**	0.34 (0.31)
		Summer	Camas	0.47 (0.45)	−**0.86 (0.42)**	−0.01 (0.54)	−**1.04 (0.50)**	−0.50 (0.45)
			Cedar Gulch	−0.02 (0.29)	−0.05 (0.29)	**1.28 (0.33)**	−0.28 (0.29)	0.47 (0.31)

Abbreviations: AC, aerial concealment (%); CP, crude protein (%); D2B, distance to burrow (m); DTR, diurnal temperature range; MT, total monoterpene concentration (scaled).

Bolded values have 95% confidence intervals that do not overlap with zero. Tables with all the models tested are in Appendix S1, Tables S9–S28.

Consistent with our second hypothesis, we documented a greater influence of food on foraging decisions (bites) than general habitat use (pellets) (Figure 3). Food appeared as the most highly supported model in 5 of 8 comparisons based on bite data, and only 3 of 8 comparisons based on pellet data (Table 2). Individual food variables were strongly influential in 3 of 8 comparisons of both bites and pellets (Table 3).

There was some support for a greater influence of food on the intensity of use at the microsite than at the patch scale (H3), with food as the most highly supported model in 4 of 8 comparisons (and competitive for 1) at the microsite scale and for 2 of 8 (and competitive in 1) at the patch scale. However, individual food variables were only influential in 2 of 8 microsite comparisons compared to 4 of 8 at the patch scale. We did find support that security was more influential at the patch scale (Figure 3) because security was the most highly supported model in 2 of 8 (and competitive in 1) comparisons at the microsite scale and 5 of 8 (competitive in 1) at the patch scale, and individual security variables were highly influential in 3 of 8 patch‐scale and 6 of 8 microsite comparisons.

In contrast to our expectations that food would be more influential during winter than summer (H4), we found some evidence for the opposite. Individual food variables were more influential in 4 of 8 comparisons for summer, but only 2 of 8 comparisons for winter, and food was the most highly supported model more often in summer (5 of 8 comparisons) than winter (2 of 8 comparisons). We did not find support for our expectation that thermal refuge would be more influential in summer than winter (H4). The thermal refuge was the most supported model in 3 of 8 winter (1 competitive) comparisons, and only 1 of 8 (with 2 competitive) summer comparisons. Similarly, individual thermal variables were influential in 6 of 8 comparisons for winter and 2 of 8 comparisons for summer. However, there was stronger evidence that security was even more influential than thermal refuge in winter (5 of 8 comparisons) compared to summer (2 of 8 comparisons, 2 more competitive), and 6 of 8 security variables in winter compared to 3 of 8 in summer.

Based on model‐averaged parameter estimates (Table 3), covariates influenced use by pygmy rabbits mostly as expected. The use of microsites and patches increased with crude protein (Figure 3, Table 3). Total monoterpene content of plants had both positive and negative effects on the use of microsites and patches, though model‐averaged parameter estimates always had confidence intervals overlapping 0 except for general use of patches in winter at Cedar Gulch (positive) and general use of patches in summer at Camas (negative). Both microsites and patches that provided more aerial concealment and were closer to an active burrow had more foraging and general use (Figure 3, Table 3). DTR exerted a variable effect on use at both scales, with model‐averaged parameter estimates always overlapping zero (Table 3).

## 4 DISCUSSION

Even within a relatively simple ecological system consisting of a sagebrush specialist herbivore residing in landscapes dominated by sagebrush, habitat use by pygmy rabbits varied with the functional properties provided (food, security, or thermal refuge), between landscapes (Camas or Cedar Gulch), by functional use (foraging or general use), across spatial scale (microsite or patch), and between seasons (winter or summer), and not always in the ways we expected. Our results emphasize the role of habitat features in providing multiple functional values to wildlife, but also the need for improving our ability to understand and measure how habitat features provide these values over multiple spatial scales and seasons, especially in even more complex ecological systems.

As we expected (H1), habitat use varied between our study sites that differed in phytochemical and structural characteristics of sagebrush. Food was more strongly associated with the use of microsites and patches by pygmy rabbits at Camas than Cedar Gulch (according to one of our measures). Sagebrush at Camas had lower levels of crude protein and higher levels of monoterpenes than did Cedar Gulch (Olsoy et al., 2020; Ulappa et al., 2014). In captive experiments, pygmy rabbits selected diets with lower fiber (thus higher crude protein) and lower levels of the monoterpene 1,8‐cineole found in sagebrush (Camp et al., 2015). Therefore, wild pygmy rabbits at Camas likely made a concerted effort to find sagebrush plants with more nutritious and less toxic leaves. That crude protein was a more consistent predictor of microsite and patch use by pygmy rabbits than monoterpenes conforms to previous findings that dietary specialists may rely on physiological mechanisms to tolerate ingested PSMs rather than behavioral avoidance (Forbey & Foley, 2009; Nobler et al., 2019). In addition, the type and concentration of individual monoterpenes, rather than total monoterpenes (which we used in this study), might be more predictive for the use of sagebrush. This pattern has been documented in wild (Ulappa et al., 2014) and captive (Nobler et al., 2019) pygmy rabbits and greater sage‐grouse (Frye et al., 2013).

Pygmy rabbits, like other lagomorphs (MacArthur & Wang, 1973; Marai et al., 2002), are more sensitive to extremely high temperatures than low temperatures, especially in our study sites (Milling et al., 2017). However, in all seasons and sites, the model‐averaged confidence intervals for DTR overlapped zero, suggesting that the effect of DTR on habitat use was modest in both landscapes. During the years studied, Cedar Gulch had a warmer June and higher annual temperature variability than both its 22‐year average and temperatures at Camas (National Weather Service, 2022). Despite this potential increased thermal stress, pygmy rabbits at Cedar Gulch did not choose microsites or patches based on DTR. Instead, the use of sites closer to burrows, which was stronger at Cedar Gulch than Camas, may allow rabbits to gain both thermal and security cover in all seasons.

The influence of security was higher at Cedar Gulch than at Camas, perhaps due to the shorter stature and sparser aerial cover of sagebrush plants at Cedar Gulch in both on‐ and off‐mound patches (Olsoy et al., 2018). Such sparse vegetation would make concealment cover of high importance to foraging herbivores. These different structural landscape patterns at Camas and Cedar Gulch might also explain why distance to burrow was more influential in predicting which plants pygmy rabbits browsed at Cedar Gulch. Sagebrush patches at Cedar Gulch were morphologically distinct, with on‐mound patches of tall, dense sagebrush easily distinguished from the matrix of dwarf sagebrush that provided poor aerial concealment. In contrast, on‐mound patches of Wyoming big sagebrush at Camas were surrounded by similarly tall off‐mound Wyoming big sagebrush that provided higher security cover (Olsoy et al., 2018), simultaneously providing cooler temperatures and safer conditions to find diets higher in crude protein.

Measuring functional properties of habitat and multiple distinct uses of habitat (e.g., foraging, hiding, and thermoregulating) might help disentangle why herbivores select habitats and habitat features. Linking selection for a habitat feature to the functional use of that feature is under‐studied, in part because types of use cannot always be distinguished, especially from traditional very high‐frequency (VHF) and GPS telemetry data. We provide an example of how food traits predicted the extent of a distinct behavior—foraging on an individual shrub (number of bites) and across a landscape (the presence of bites in patches). As expected, food tended to be more influential when predicting the plants and patches browsed by pygmy rabbits than those used for general use. More distinct measures of behaviors are required to distinguish habitat traits that support unique behaviors compared to multiple activities.

Scale is often vital to properly infer the relative influence of food, security, and thermal refuge in habitat selection studies (Holling, 1992; Rettie & Messier, 2000). Pygmy rabbits used greater aerial concealment closer to burrows at the patch scale more often than at the microsite, which supported our hypothesis (H3). This finding suggests that risk‐based limiting factors may act more strongly on broad scales (Hebblewhite & Merrill, 2009; Rettie & Messier, 2000). In support, captive pygmy rabbits consistently chose food patches with higher levels of concealment, regardless of an immediate threat of predators (Camp et al., 2017; Crowell et al., 2016). However, in a natural landscape, less use of unsafe patches could indicate that pygmy rabbits that used those sites were depredated (Messinger et al., 2019). In contrast, food traits were important at both microsite and patch scales for pygmy rabbits. Similarly, Frye et al. (2013) found that the greater sage‐grouse, another vertebrate herbivore that is a sagebrush‐obligate, selected for phytochemicals at both the microsite and patch scales. Taken together, the strong influence of food on selection across scales by sagebrush herbivores suggests that forage quality should be included in habitat selection studies along with structural traits in efforts to manage herbivores across landscapes.

Despite known seasonal changes in biomass availability and nutritional quality of vegetation used for food and security and temperature fluctuations, the relative influence of each functional property on how pygmy rabbits used microsites and patches varied less than expected between winter and summer. We expected crude protein to be more influential when consuming bites of forage in winter when crude protein in sagebrush averages three times higher than in senescent grasses and forbs than in summer when nutritional quality is less divergent (Thines et al., 2004). We also expected thermal variables to be more influential in the use of microsites and patches of habitat use in summer than winter both because our study sites experienced extreme (20–22°C) differences in seasonal ambient temperatures, and because pygmy rabbits are more sensitive to very high temperatures than very low temperatures, as evidenced by their rest site selection during summer in a previous study at Cedar Gulch (Milling et al., 2017). The lower importance of thermal variables might reflect in part the year‐round predation risk (5%–40% predation mortality per month; Crawford et al., 2010; Sanchez, 2007) where consistent use of vegetative cover and maintaining proximity to burrows provide both concealments from predation and thermal refuge.

Our results demonstrated complex patterns of function‐, context‐, and scale‐dependent habitat use by pygmy rabbits. Despite constraints imposed on the use of habitat by central‐place foraging behaviors, small body size, and vulnerability to a diverse suite of predators, we found that pygmy rabbits used microsites and patches that provided relatively different functional properties among seasons and landscapes. Improving our understanding of the ecological factors that underpin habitat selection and space use by herbivores requires that we go beyond measuring only habitat structure and focus on measuring or predicting the specific functional properties that habitat features provide to animals at multiple scales. Broader application of such approaches will not only advance knowledge about the ecology of species and their habitats but will also facilitate better predictions of how species might respond to natural and managed changes in habitats at both small and large spatiotemporal scales.

## CONFLICT OF INTEREST

The authors declare no conflicts of interest.

## AUTHOR CONTRIBUTIONS

**Peter J. Olsoy:** Data curation (equal); Formal analysis (lead); Investigation (supporting); Methodology (equal); Visualization (lead); Writing – original draft (lead); Writing – review & editing (equal). **Charlotte R. Milling:** Data curation (equal); Investigation (equal); Methodology (equal); Writing – review & editing (supporting). **Jordan D. Nobler:** Data curation (equal); Investigation (equal); Methodology (equal); Writing – review & editing (supporting). **Meghan J. Camp:** Investigation (equal); Methodology (equal); Writing – review & editing (supporting). **Lisa A. Shipley:** Conceptualization (equal); Funding acquisition (equal); Investigation (equal); Resources (equal); Supervision (equal); Writing – original draft (supporting); Writing – review & editing (equal). **Jennifer S. Forbey:** Conceptualization (equal); Funding acquisition (equal); Investigation (equal); Resources (equal); Supervision (equal); Writing – review & editing (equal). **Janet L. Rachlow:** Conceptualization (equal); Funding acquisition (equal); Investigation (equal); Resources (equal); Supervision (equal); Writing – review & editing (equal). **Daniel H. Thornton:** Conceptualization (equal); Formal analysis (supporting); Methodology (supporting); Resources (equal); Supervision (equal); Writing – original draft (supporting); Writing – review & editing (equal).

## Supporting information

Supplementary Material

## Data Availability

The data that support the findings of this study are openly available from the University of Idaho at http://doi.org/10.7923/RK1C‐JZ69 (Olsoy et al., 2022).
