# Supplementary material for: Food quality, security, and thermal refuge influence the use of microsites and patches by pygmy rabbits (Brachylagus idahoensis) across landscapes and seasons
Source: Ecol Evol. 2022 May 13;12(5):e8892. doi: 10.1002/ece3.8892 (PMC9106561; doi:10.1002/ece3.8892)
Supplement: Supplementary file 1 — Supplementary Material [file ECE3-12-e8892-s001.docx]

**Appendix S1. Correlation matrices, and full AIC_c_ tables for all model sets.**

Table S1. Cedar Gulch – Winter – Microsite-scale correlation matrix

|  | AC | D2B | CP | MT | DTR |
| --- | --- | --- | --- | --- | --- |
| AC | 1.00 | -0.20 | 0.16 | 0.39 | -0.54 |
| D2B | - | 1.00 | 0.01 | -0.07 | 0.12 |
| CP | - | - | 1.00 | 0.34 | -0.12 |
| MT | - | - | - | 1.00 | -0.33 |
| DTR | - | - | - | - | 1.00 |

Table S2. Camas – Winter – Microsite-scale correlation matrix

|  | AC | D2B | CP | MT | DTR |
| --- | --- | --- | --- | --- | --- |
| AC | 1.00 | -0.07 | 0.12 | 0.02 | -0.19 |
| D2B | - | 1.00 | -0.31 | -0.22 | 0.03 |
| CP | - | - | 1.00 | 0.60 | 0.02 |
| MT | - | - | - | 1.00 | -0.05 |
| DTR | - | - | - | - | 1.00 |

Table S3. Cedar Gulch – Summer – Microsite-scale correlation matrix

|  | AC | D2B | CP | MT | DTR |
| --- | --- | --- | --- | --- | --- |
| AC | 1.00 | -0.22 | 0.16 | 0.12 | -0.49 |
| D2B | - | 1.00 | 0.06 | -0.02 | 0.05 |
| CP | - | - | 1.00 | 0.08 | -0.03 |
| MT | - | - | - | 1.00 | -0.07 |
| DTR | - | - | - | - | 1.00 |

Table S4. Camas – Summer – Microsite-scale correlation matrix

|  | AC | D2B | CP | MT | DTR |
| --- | --- | --- | --- | --- | --- |
| AC | 1.00 | -0.11 | 0.09 | 0.20 | -0.31 |
| D2B | - | 1.00 | -0.23 | -0.40 | 0.15 |
| CP | - | - | 1.00 | 0.45 | -0.09 |
| MT | - | - | - | 1.00 | -0.20 |
| DTR | - | - | - | - | 1.00 |

Table S5. Cedar Gulch – Winter – Patch-scale correlation matrix

|  | AC | D2B | CP | MT | DTR |
| --- | --- | --- | --- | --- | --- |
| AC | 1.00 | -0.28 | 0.19 | 0.34 | -0.66 |
| D2B | - | 1.00 | 0.01 | 0.00 | 0.09 |
| CP | - | - | 1.00 | 0.35 | -0.25 |
| MT | - | - | - | 1.00 | -0.43 |
| DTR | - | - | - | - | 1.00 |

Table S6. Camas – Winter – Patch-scale correlation matrix

|  | AC | D2B | CP | MT | DTR |
| --- | --- | --- | --- | --- | --- |
| AC | 1.00 | -0.16 | 0.24 | 0.26 | -0.32 |
| D2B | - | 1.00 | -0.41 | -0.31 | 0.07 |
| CP | - | - | 1.00 | 0.80 | 0.05 |
| MT | - | - | - | 1.00 | -0.01 |
| DTR | - | - | - | - | 1.00 |

Table S7. Cedar Gulch – Summer – Patch-scale correlation matrix

|  | AC | D2B | CP | MT | DTR |
| --- | --- | --- | --- | --- | --- |
| AC | 1.00 | -0.22 | 0.13 | 0.00 | -0.52 |
| D2B | - | 1.00 | 0.04 | -0.04 | 0.03 |
| CP | - | - | 1.00 | 0.07 | 0.00 |
| MT | - | - | - | 1.00 | -0.04 |
| DTR | - | - | - | - | 1.00 |

Table S8. Camas – Summer – Patch-scale correlation matrix

|  | AC | D2B | CP | MT | DTR |
| --- | --- | --- | --- | --- | --- |
| AC | 1.00 | -0.10 | 0.18 | 0.28 | -0.48 |
| D2B | - | 1.00 | -0.25 | -0.38 | 0.14 |
| CP | - | - | 1.00 | 0.52 | -0.10 |
| MT | - | - | - | 1.00 | -0.23 |
| DTR | - | - | - | - | 1.00 |

Table S9. Full AICc table for microsite-scale bites – Cedar Gulch – Winter

| Model | (Int) | AC | CP | D2B | DTR | MT | df | logLik | AICc | delta | weights |
| --- | --- | --- | --- | --- | --- | --- | --- | --- | --- | --- | --- |
| D2B | -2.69 |  |  | -0.196 |  |  | 4 | -127.96 | 264.1 | 0.0 | 0.287 |
| THERMAL | -2.98 |  |  | -0.214 | 0.143 |  | 5 | -127.43 | 265.2 | 1.1 | 0.166 |
| SECURITY | -2.79 | 1.401 |  | -0.185 |  |  | 5 | -127.44 | 265.2 | 1.1 | 0.166 |
| SECURITY+THERMAL | -3.41 | 1.731 |  | -0.194 | 0.179 |  | 6 | -126.66 | 265.7 | 1.6 | 0.129 |
| FOOD+SECURITY | -4.89 | 1.727 | 0.189 | -0.186 |  | 0.532 | 7 | -126.42 | 267.4 | 3.3 | 0.055 |
| NULL | -8.73 |  |  |  |  |  | 3 | -130.88 | 267.9 | 3.8 | 0.043 |
| FOOD+THERMAL | -5.56 |  | 0.215 | -0.206 | 0.108 | 0.219 | 7 | -126.97 | 268.5 | 4.4 | 0.032 |
| GLOBAL | -5.63 | 1.860 | 0.204 | -0.190 | 0.127 | 0.343 | 8 | -126.05 | 268.8 | 4.7 | 0.027 |
| DTR | -9.03 |  |  |  | 0.127 |  | 4 | -130.42 | 269.0 | 4.9 | 0.025 |
| CP | -12.12 |  | 0.251 |  |  |  | 4 | -130.42 | 269.1 | 5.0 | 0.024 |
| AC | -8.96 | 1.185 |  |  |  |  | 4 | -130.48 | 269.2 | 5.1 | 0.022 |
| MT | -8.69 |  |  |  |  | 0.128 | 4 | -130.84 | 269.9 | 5.8 | 0.016 |
| FOOD | -12.08 |  | 0.249 |  |  | 0.116 | 5 | -130.39 | 271.1 | 7.0 | 0.009 |

Table S10. Full AICc table for microsite-scale bites – Camas – Winter

| Model | (Int) | AC | CP | D2B | DTR | MT | df | logLik | AICc | delta | weights |
| --- | --- | --- | --- | --- | --- | --- | --- | --- | --- | --- | --- |
| CP | -11.30 |  | 0.513 |  |  |  | 4 | -105.64 | 219.6 | 0.0 | 0.175 |
| DTR | -7.33 |  |  |  | -0.238 |  | 4 | -105.84 | 220.0 | 0.4 | 0.143 |
| THERMAL | -4.54 |  |  | -0.040 | -0.228 |  | 5 | -104.92 | 220.3 | 0.7 | 0.124 |
| SECURITY+THERMAL | -5.36 | 1.968 |  | -0.041 | -0.235 |  | 6 | -104.20 | 221.1 | 1.5 | 0.083 |
| FOOD | -11.63 |  | 0.549 |  |  | -0.025 | 5 | -105.50 | 221.5 | 1.9 | 0.068 |
| FOOD+SECURITY | -10.44 | 2.727 | 0.590 | -0.037 |  | 0.055 | 7 | -103.30 | 221.5 | 1.9 | 0.068 |
| NULL | -6.82 |  |  |  |  |  | 3 | -107.68 | 221.6 | 2.0 | 0.064 |
| D2B | -3.84 |  |  | -0.043 |  |  | 4 | -106.63 | 221.6 | 2.0 | 0.064 |
| FOOD+THERMAL | -8.13 |  | 0.403 | -0.035 | -0.178 | 0.024 | 7 | -103.66 | 222.2 | 2.6 | 0.048 |
| GLOBAL | -9.72 | 2.427 | 0.469 | -0.036 | -0.166 | 0.064 | 8 | -102.52 | 222.2 | 2.6 | 0.048 |
| AC | -7.58 | 1.868 |  |  |  |  | 4 | -107.01 | 222.3 | 2.7 | 0.045 |
| SECURITY | -4.55 | 1.847 |  | -0.043 |  |  | 5 | -105.94 | 222.4 | 2.8 | 0.043 |
| MT | -6.69 |  |  |  |  | 0.282 | 4 | -107.54 | 223.4 | 3.8 | 0.026 |

Table S11. Full AICc table for microsite-scale bites – Cedar Gulch – Summer

| Model | (Int) | AC | CP | D2B | DTR | MT | df | logLik | AICc | delta | weights |
| --- | --- | --- | --- | --- | --- | --- | --- | --- | --- | --- | --- |
| FOOD+THERMAL | -13.04 |  | 0.827 | -0.044 | -0.243 | -0.935 | 7 | -59.07 | 132.8 | 0.0 | 0.549 |
| GLOBAL | -14.06 | 1.310 | 0.844 | -0.038 | -0.202 | -0.975 | 8 | -58.84 | 134.6 | 1.8 | 0.223 |
| FOOD+SECURITY | -12.38 | 3.215 | 0.674 | -0.031 |  | -0.867 | 7 | -60.49 | 135.7 | 2.9 | 0.129 |
| CP | -11.80 |  | 0.441 |  |  |  | 4 | -65.75 | 139.7 | 6.9 | 0.017 |
| AC | -7.21 | 3.045 |  |  |  |  | 4 | -65.78 | 139.8 | 7.0 | 0.017 |
| NULL | -6.63 |  |  |  |  |  | 3 | -66.88 | 139.9 | 7.1 | 0.016 |
| D2B | -4.95 |  |  | -0.035 |  |  | 4 | -66.33 | 140.9 | 8.1 | 0.010 |
| SECURITY | -5.44 | 2.789 |  | -0.030 |  |  | 5 | -65.29 | 140.9 | 8.1 | 0.010 |
| DTR | -6.39 |  |  |  | -0.141 |  | 4 | -66.42 | 141.1 | 8.3 | 0.009 |
| FOOD | -12.17 |  | 0.490 |  |  | -0.212 | 5 | -65.70 | 141.8 | 9.0 | 0.006 |
| THERMAL | -4.29 |  |  | -0.038 | -0.151 |  | 5 | -65.70 | 141.8 | 9.0 | 0.006 |
| MT | -6.66 |  |  |  |  | 0.112 | 4 | -66.86 | 142.0 | 9.2 | 0.006 |
| SECURITY+THERMAL | -4.91 | 2.242 |  | -0.033 | -0.075 |  | 6 | -65.14 | 142.8 | 10.0 | 0.004 |

Table S12. Full AICc table for microsite-scale bites – Camas – Summer

| Model | (Int) | AC | CP | D2B | DTR | MT | df | logLik | AICc | delta | weights |
| --- | --- | --- | --- | --- | --- | --- | --- | --- | --- | --- | --- |
| MT | -9.48 |  |  |  |  | 1.637 | 4 | -31.33 | 70.9 | 0.0 | 0.289 |
| FOOD | -17.05 |  | 0.711 |  |  | 1.224 | 5 | -30.54 | 71.5 | 0.6 | 0.214 |
| CP | -16.74 |  | 0.830 |  |  |  | 4 | -31.78 | 71.8 | 0.9 | 0.184 |
| NULL | -7.12 |  |  |  |  |  | 3 | -33.74 | 73.6 | 2.7 | 0.075 |
| FOOD+THERMAL | -21.43 |  | 0.895 | 0.035 | 0.148 | 1.357 | 7 | -29.70 | 74.1 | 3.2 | 0.058 |
| FOOD+SECURITY | -18.46 | -0.667 | 0.685 | 0.034 |  | 1.461 | 7 | -29.98 | 74.7 | 3.8 | 0.043 |
| AC | -6.80 | -0.670 |  |  |  |  | 4 | -33.53 | 75.3 | 4.4 | 0.032 |
| DTR | -7.16 |  |  |  | 0.077 |  | 4 | -33.58 | 75.4 | 4.5 | 0.030 |
| D2B | -7.46 |  |  | 0.006 |  |  | 4 | -33.71 | 75.7 | 4.8 | 0.026 |
| GLOBAL | -20.78 | -0.452 | 0.848 | 0.037 | 0.134 | 1.421 | 8 | -29.65 | 76.2 | 5.3 | 0.020 |
| SECURITY | -7.15 | -0.669 |  | 0.006 |  |  | 5 | -33.50 | 77.4 | 6.5 | 0.011 |
| THERMAL | -7.45 |  |  | 0.005 | 0.075 |  | 5 | -33.57 | 77.5 | 6.6 | 0.011 |
| SECURITY+THERMAL | -7.19 | -0.572 |  | 0.005 | 0.058 |  | 6 | -33.41 | 79.4 | 8.5 | 0.004 |

Table S13. Full AICc table for microsite-scale pellets – Cedar Gulch – Winter

| Model | (Int) | AC | CP | D2B | DTR | MT | df | logLik | AICc | delta | weights |
| --- | --- | --- | --- | --- | --- | --- | --- | --- | --- | --- | --- |
| THERMAL | -0.36 |  |  | -0.085 | -0.155 |  | 5 | -326.76 | 663.8 | 0.0 | 0.388 |
| SECURITY+THERMAL | -0.49 | 0.915 |  | -0.080 | -0.145 |  | 6 | -326.11 | 664.7 | 0.9 | 0.247 |
| FOOD+THERMAL | -3.21 |  | 0.213 | -0.084 | -0.165 | -0.018 | 7 | -325.83 | 666.2 | 2.4 | 0.117 |
| GLOBAL | -3.60 | 0.974 | 0.228 | -0.079 | -0.152 | -0.045 | 8 | -325.06 | 666.9 | 3.1 | 0.082 |
| D2B | -1.02 |  |  | -0.085 |  |  | 4 | -329.60 | 667.4 | 3.6 | 0.064 |
| SECURITY | -0.96 | 1.161 |  | -0.080 |  |  | 5 | -328.61 | 667.5 | 3.7 | 0.061 |
| FOOD+SECURITY | -3.66 | 1.208 | 0.203 | -0.080 |  | 0.005 | 7 | -327.83 | 670.2 | 6.4 | 0.016 |
| DTR | -5.76 |  |  |  | -0.130 |  | 4 | -331.39 | 671.0 | 7.2 | 0.011 |
| NULL | -6.23 |  |  |  |  |  | 3 | -333.18 | 672.5 | 8.7 | 0.005 |
| CP | -8.77 |  | 0.193 |  |  |  | 4 | -332.51 | 673.2 | 9.4 | 0.004 |
| AC | -6.13 | 0.918 |  |  |  |  | 4 | -332.57 | 673.3 | 9.5 | 0.003 |
| MT | -6.24 |  |  |  |  | -0.014 | 4 | -333.18 | 674.6 | 10.8 | 0.002 |
| FOOD | -8.93 |  | 0.202 |  |  | -0.066 | 5 | -332.46 | 675.2 | 11.4 | 0.001 |

Table S14. Full AICc table for microsite-scale pellets – Camas – Winter

| Model | (Int) | AC | CP | D2B | DTR | MT | df | logLik | AICc | delta | weights |
| --- | --- | --- | --- | --- | --- | --- | --- | --- | --- | --- | --- |
| CP | -8.38 |  | 0.841 |  |  |  | 4 | -142.85 | 294.0 | 0.0 | 0.664 |
| FOOD | -8.35 |  | 0.838 |  |  | 0.010 | 5 | -142.85 | 296.2 | 2.2 | 0.221 |
| FOOD+THERMAL | -8.78 |  | 0.891 | -0.003 | 0.154 | 0.032 |  | -142.00 | 298.8 | 4.8 | 0.060 |
| FOOD+SECURITY | -8.70 | -0.616 | 0.865 | 0.006 |  | -0.011 | 7 | -142.67 | 300.2 | 6.2 | 0.030 |
| GLOBAL | -8.58 | -0.832 | 0.906 | -0.002 | 0.162 | 0.017 | 8 | -141.80 | 300.8 | 6.8 | 0.022 |
| MT | 0.08 |  |  |  |  | 1.105 | 4 | -149.32 | 306.9 | 12.9 | 0.001 |
| NULL | 0.10 |  |  |  |  |  | 3 | -151.29 | 308.8 | 14.8 | 0.000 |
| D2B | 0.68 |  |  | -0.024 |  |  | 4 | -150.57 | 309.4 | 15.4 | 0.000 |
| AC | 0.41 | -0.528 |  |  |  |  | 4 | -151.25 | 310.8 | 16.8 | 0.000 |
| DTR | 0.09 |  |  |  | 0.026 |  | 4 | -151.27 | 310.9 | 16.9 | 0.000 |
| THERMAL | 0.83 |  |  | -0.028 | 0.097 |  | 5 | -150.31 | 311.1 | 17.1 | 0.000 |
| SECURITY | 0.85 | -0.307 |  | -0.023 |  |  | 5 | -150.55 | 311.6 | 17.6 | 0.000 |
| SECURITY+THERMAL | 0.94 | -0.187 |  | -0.027 | 0.096 |  | 6 | -150.30 | 313.3 | 19.3 | 0.000 |

Table S15. Full AICc table for microsite-scale pellets – Cedar Gulch – Summer

| Model | (Int) | AC | CP | D2B | DTR | MT | df | logLik | AICc | delta | weights |
| --- | --- | --- | --- | --- | --- | --- | --- | --- | --- | --- | --- |
| SECURITY+THERMAL | -0.41 | 3.908 |  | -0.040 | 0.098 |  | 6 | -331.23 | 675.0 | 0.0 | 0.279 |
| FOOD+SECURITY | -3.37 | 3.632 | 0.230 | -0.038 |  | -0.227 | 7 | -330.15 | 675.0 | 0.0 | 0.279 |
| GLOBAL | -3.26 | 4.035 | 0.210 | -0.039 | 0.080 | -0.204 | 8 | -329.22 | 675.4 | 0.4 | 0.228 |
| SECURITY | -0.22 | 3.430 |  | -0.040 |  |  | 5 | -332.59 | 675.6 | 0.6 | 0.207 |
| AC | -1.79 | 3.910 |  |  |  |  | 4 | -337.07 | 682.4 | 7.4 | 0.007 |
| D2B | 1.28 |  |  | -0.053 |  |  | 4 | -340.07 | 688.4 | 13.4 | 0.000 |
| THERMAL | 1.27 |  |  | -0.054 | 0.035 |  | 5 | -339.92 | 690.2 | 15.2 | 0.000 |
| FOOD+THERMAL | -1.45 |  | 0.201 | -0.052 | 0.019 | -0.016 | 7 | -338.58 | 691.9 | 16.9 | 0.000 |
| CP | -3.35 |  | 0.203 |  |  |  | 4 | -344.63 | 697.5 | 22.5 | 0.000 |
| NULL | -0.66 |  |  |  |  |  | 3 | -345.96 | 698.1 | 23.1 | 0.000 |
| FOOD | -3.33 |  | 0.200 |  |  | 0.062 | 5 | -344.60 | 699.6 | 24.6 | 0.000 |
| DTR | -0.69 |  |  |  | 0.027 |  | 4 | -345.87 | 700.0 | 25.0 | 0.000 |
| MT | -0.67 |  |  |  |  | 0.098 | 4 | -345.88 | 700.0 | 25.0 | 0.000 |

Table S16. Full AICc table for microsite-scale pellets – Camas – Summer

| Model | (Int) | AC | CP | D2B | DTR | MT | df | logLik | AICc | delta | weights |
| --- | --- | --- | --- | --- | --- | --- | --- | --- | --- | --- | --- |
| NULL | -6.96 |  |  |  |  |  | 3 | -40.23 | 86.6 | 0.0 | 0.215 |
| MT | -7.48 |  |  |  |  | 0.869 | 4 | -39.38 | 87.0 | 0.4 | 0.176 |
| D2B | -4.88 |  |  | -0.026 |  |  | 4 | -39.75 | 87.8 | 1.2 | 0.118 |
| FOOD | -3.48 |  | -0.416 |  |  | 1.105 | 5 | -38.95 | 88.3 | 1.7 | 0.092 |
| DTR | -6.87 |  |  |  | -0.087 |  | 4 | -40.05 | 88.4 | 1.8 | 0.088 |
| CP | -5.36 |  | -0.171 |  |  |  | 4 | -40.14 | 88.5 | 1.9 | 0.083 |
| AC | -6.71 | -0.774 |  |  |  |  | 4 | -40.17 | 88.6 | 2.0 | 0.079 |
| THERMAL | -4.72 |  |  | -0.026 | -0.080 |  | 5 | -39.58 | 89.5 | 2.9 | 0.051 |
| SECURITY | -4.61 | -0.767 |  | -0.027 |  |  | 5 | -39.68 | 89.7 | 3.1 | 0.046 |
| SECURITY+THERMAL | -4.34 | -0.915 |  | -0.026 | -0.091 |  | 6 | -39.47 | 91.5 | 4.9 | 0.019 |
| FOOD+THERMAL | -1.08 |  | -0.516 | -0.020 | -0.103 | 0.926 | 7 | -38.59 | 91.9 | 5.3 | 0.015 |
| FOOD+SECURITY | -2.05 | -0.387 | -0.410 | -0.018 |  | 0.951 | 7 | -38.78 | 92.3 | 5.7 | 0.012 |
| GLOBAL | -1.04 | -0.431 | -0.496 | -0.020 | -0.104 | 0.894 | 8 | -38.57 | 94.1 | 7.5 | 0.005 |

Table S17. Full AICc table for patch-scale bites – Camas – Winter

| Model | (Int) | CP | D2B | MT | AC | DTR | df | logLik | AICc | delta | weights |
| --- | --- | --- | --- | --- | --- | --- | --- | --- | --- | --- | --- |
| SAFETY | -4.228 |  | -0.034 |  | 2.693 |  | 3 | -20.18 | 46.8 | 0 | 0.238 |
| THERMAL | 62.840 |  | -0.035 |  |  | -2.894 | 3 | -20.49 | 47.5 | 0.7 | 0.168 |
| D2B | -2.170 |  | -0.037 |  |  |  | 2 | -21.74 | 47.7 | 0.9 | 0.152 |
| CP | -11.660 | 0.733 |  |  |  |  | 2 | -22.01 | 48.3 | 1.5 | 0.112 |
| SAFETY+THERMAL | 12.630 |  | -0.034 |  | 2.185 | -0.733 | 4 | -20.15 | 49.1 | 2.3 | 0.075 |
| FOOD+SAFETY | -10.730 | 0.634 | -0.029 | -0.548 | 2.392 |  | 5 | -18.98 | 49.2 | 2.4 | 0.072 |
| FOOD+THERMAL | 43.760 | 0.615 | -0.030 | -0.417 |  | -2.339 | 5 | -19.25 | 49.8 | 3 | 0.053 |
| FOOD | -12.730 | 0.843 |  | -0.271 |  |  | 3 | -21.90 | 50.3 | 3.5 | 0.041 |
| AC | -6.240 |  |  |  | 3.222 |  | 2 | -23.17 | 50.6 | 3.8 | 0.036 |
| GLOBAL | -2.964 | 0.630 | -0.029 | -0.550 | 2.166 | -0.336 | 6 | -18.97 | 51.8 | 5 | 0.020 |
| DTR | 67.910 |  |  |  |  | -3.195 | 2 | -23.92 | 52.1 | 5.3 | 0.017 |
| MT | -4.072 |  |  | 0.615 |  |  | 2 | -24.48 | 53.2 | 6.4 | 0.010 |
| NULL | -3.924 |  |  |  |  |  | 1 | -25.82 | 53.7 | 6.9 | 0.008 |

Table S18. Full AICc table for patch-scale bites – Cedar Gulch – Winter

| Model | (Int) | CP | D2B | MT | AC | DTR | df | logLik | AICc | delta | weights |
| --- | --- | --- | --- | --- | --- | --- | --- | --- | --- | --- | --- |
| FOOD+SAFETY | 7.474 | -0.927 | -0.135 | 1.149 | 6.429 |  | 5 | -17.26 | 45.4 | 0.0 | 0.414 |
| SAFETY | -4.981 |  | -0.125 |  | 7.080 |  | 3 | -19.801 | 45.9 | 0.5 | 0.322 |
| GLOBAL | -11.010 | -0.951 | -0.132 | 1.322 | 7.566 | 0.686 | 6 | -17.142 | 47.5 | 2.1 | 0.145 |
| SAFETY+THERMAL | -4.563 |  | -0.125 |  | 7.053 | -0.015 | 4 | -19.801 | 48.2 | 2.8 | 0.102 |
| FOOD+THERMAL | 51.520 | -0.842 | -0.135 | 1.475 |  | -1.591 | 5 | -20.806 | 52.4 | 7.0 | 0.013 |
| THERMAL | 58.670 |  | -0.128 |  |  | -2.277 | 3 | -24.243 | 54.8 | 9.4 | 0.004 |
| D2B | -1.290 |  | -0.133 |  |  |  | 2 | -28.066 | 60.3 | 14.9 | 0.000 |
| AC | -7.405 |  |  |  | 6.203 |  | 2 | -31.03 | 66.2 | 20.8 | 0.000 |
| DTR | 50.360 |  |  |  |  | -2.076 | 2 | -36.128 | 76.4 | 31.0 | 0.000 |
| FOOD | 1.848 | -0.478 |  | 0.875 |  |  | 3 | -37.532 | 81.4 | 36.0 | 0.000 |
| MT | -4.398 |  |  | 0.684 |  |  | 2 | -39.013 | 82.2 | 36.8 | 0.000 |
| NULL | -4.329 |  |  |  |  |  | 1 | -41.622 | 85.3 | 39.9 | 0.000 |
| CP | -1.134 | -0.244 |  |  |  |  | 2 | -41.183 | 86.5 | 41.1 | 0.000 |

Table S19. Full AICc table for patch-scale bites – Camas – Summer

| Model | (Int) | CP | D2B | MT | AC | DTR | df | logLik | AICc | delta | weights |
| --- | --- | --- | --- | --- | --- | --- | --- | --- | --- | --- | --- |
| NULL | -4.940 |  |  |  |  |  | 1 | -19.008 | 40.1 | 0.0 | 0.188 |
| FOOD | -13.490 | 0.772 |  | -0.885 |  |  | 3 | -17.042 | 40.5 | 0.4 | 0.154 |
| CP | -9.294 | 0.396 |  |  |  |  | 2 | -18.322 | 40.8 | 0.7 | 0.132 |
| AC | -4.407 |  |  |  | -0.981 |  | 2 | -18.742 | 41.7 | 1.6 | 0.084 |
| MT | -4.962 |  |  | -0.255 |  |  | 2 | -18.825 | 41.8 | 1.7 | 0.080 |
| D2B | -5.387 |  | 0.007 |  |  |  | 2 | -18.846 | 41.9 | 1.8 | 0.076 |
| DTR | -22.090 |  |  |  |  | 0.466 | 2 | -18.855 | 41.9 | 1.8 | 0.076 |
| FOOD+SAFETY | -17.430 | 1.295 | 0.003 | -0.480 | -3.620 |  | 5 | -15.561 | 42.1 | 2.0 | 0.069 |
| FOOD+THERMAL | -92.710 | 1.247 | 0.002 | -0.560 |  | 2.009 | 5 | -15.817 | 42.7 | 2.6 | 0.051 |
| SAFETY | -4.786 |  | 0.005 |  | -0.807 |  | 3 | -18.686 | 43.8 | 3.7 | 0.030 |
| THERMAL | -17.680 |  | 0.006 |  |  | 0.337 | 3 | -18.774 | 43.9 | 3.8 | 0.028 |
| GLOBAL | -14.750 | 1.294 | 0.003 | -0.479 | -3.728 | -0.071 | 6 | -15.561 | 44.6 | 4.5 | 0.020 |
| SAFETY+THERMAL | 47.840 |  | 0.005 |  | -2.927 | -1.400 | 4 | -18.563 | 45.8 | 5.7 | 0.011 |

Table S20. Full AICc table for patch-scale bites – Cedar Gulch – Summer

| Model | (Int) | CP | D2B | MT | AC | DTR | df | logLik | AICc | delta | weights |
| --- | --- | --- | --- | --- | --- | --- | --- | --- | --- | --- | --- |
| CP | -11.160 | 0.495 |  |  |  |  | 2 | -30.117 | 64.4 | 0.0 | 0.300 |
| FOOD+SAFETY | -13.060 | 0.628 | -0.028 | -0.423 | 1.904 |  | 5 | -27.207 | 65.2 | 0.8 | 0.201 |
| MT | -11.210 | 0.497 |  | -0.304 |  |  | 3 | -29.713 | 65.7 | 1.3 | 0.156 |
| FOOD+THERMAL | 16.510 | 0.615 | -0.031 | -0.385 |  | -0.798 | 5 | -27.872 | 66.6 | 2.2 | 0.100 |
| GLOBAL | -34.850 | 0.642 | -0.027 | -0.417 | 2.461 | 0.598 | 6 | -27.138 | 67.4 | 3.0 | 0.067 |
| AC | -5.079 |  |  |  | 2.310 |  | 2 | -31.795 | 67.7 | 3.3 | 0.058 |
| SAFETY | -4.582 |  | -0.014 |  | 2.167 |  | 3 | -31.448 | 69.2 | 4.8 | 0.027 |
| NULL | -4.091 |  |  |  |  |  | 1 | -33.602 | 69.3 | 4.9 | 0.026 |
| DTR | 41.590 |  |  |  |  | -1.281 | 2 | -32.803 | 69.8 | 5.4 | 0.020 |
| D2B | -3.526 |  | -0.017 |  |  |  | 2 | -32.979 | 70.1 | 5.7 | 0.017 |
| THERMAL | 39.650 |  | -0.016 |  |  | -1.212 | 3 | -32.281 | 70.9 | 6.5 | 0.012 |
| SAFETY+THERMAL | -8.915 |  | -0.013 |  | 2.277 | 0.120 | 4 | -31.445 | 71.4 | 7.0 | 0.009 |
| FOOD | -3.521 |  | -0.019 | -0.288 |  |  | 3 | -32.646 | 71.6 | 7.2 | 0.008 |

Table S21. Full AICc table for patch-scale pellets – Camas – Winter

| Model | (Int) | CP | D2B | MT | AC | DTR | df | logLik | AICc | delta | weights |
| --- | --- | --- | --- | --- | --- | --- | --- | --- | --- | --- | --- |
| D2B | -1.753 |  | -0.028 |  |  |  | 2 | -30.346 | 64.9 | 0.0 | 0.433 |
| SAFETY | -1.505 |  | -0.028 |  | -0.350 |  | 3 | -30.293 | 67.1 | 2.2 | 0.144 |
| THERMAL | -8.061 |  | -0.028 |  |  | 0.280 | 3 | -30.314 | 67.1 | 2.2 | 0.144 |
| NULL | -3.204 |  |  |  |  |  | 1 | -33.65 | 69.4 | 4.5 | 0.046 |
| CP | -6.161 | 0.290 |  |  |  |  | 2 | -32.599 | 69.4 | 4.5 | 0.046 |
| SAFETY+THERMAL | -0.435 |  | -0.028 |  | -0.387 | -0.046 | 4 | -30.293 | 69.4 | 4.5 | 0.046 |
| FOOD | -9.121 | 0.586 |  | -0.610 |  |  | 3 | -31.866 | 70.2 | 5.3 | 0.031 |
| FOOD+SAFETY | -5.727 | 0.398 | -0.025 | -0.492 | -0.305 |  | 5 | -29.564 | 70.4 | 5.5 | 0.028 |
| FOOD+THERMAL | -11.750 | 0.401 | -0.025 | -0.509 |  | 0.257 | 5 | -29.573 | 70.4 | 5.5 | 0.028 |
| MT | -3.213 |  |  | 0.119 |  |  | 2 | -33.574 | 71.4 | 6.5 | 0.017 |
| AC | -3.336 |  |  |  | 0.209 |  | 2 | -33.628 | 71.5 | 6.6 | 0.016 |
| DTR | 1.558 |  |  |  |  | -0.211 | 2 | -33.629 | 71.5 | 6.6 | 0.016 |
| GLOBAL | -7.096 | 0.399 | -0.025 | -0.491 | -0.261 | 0.059 | 6 | -29.564 | 73.0 | 8.1 | 0.008 |

Table S22. Full AICc table for patch-scale pellets – Cedar Gulch – Winter

| Model | (Int) | CP | D2B | MT | AC | DTR | df | logLik | AICc | delta | weights |
| --- | --- | --- | --- | --- | --- | --- | --- | --- | --- | --- | --- |
| FOOD+SAFETY | 2.335 | -0.435 | -0.033 | 0.905 | 4.646 |  | 5 | -35.411 | 81.7 | 0.0 | 0.486 |
| GLOBAL | -26.030 | -0.481 | -0.038 | 0.874 | 6.112 | 1.079 | 6 | -34.699 | 82.6 | 0.9 | 0.310 |
| SAFETY | -3.867 |  | -0.025 |  | 5.288 |  | 3 | -39.330 | 85.0 | 3.3 | 0.093 |
| SAFETY+THERMAL | -33.180 |  | -0.030 |  | 6.678 | 1.095 | 4 | -38.556 | 85.7 | 4.0 | 0.066 |
| AC | -4.899 |  |  |  | 5.591 |  | 2 | -41.146 | 86.5 | 4.8 | 0.044 |
| FOOD+THERMAL | 32.610 | -0.261 | -0.032 | 1.278 |  | -1.167 | 5 | -42.151 | 95.1 | 13.4 | 0.001 |
| MT | -2.822 |  |  | 1.271 |  |  | 2 | -49.484 | 103.1 | 21.4 | 0.000 |
| FOOD | 0.838 | -0.277 |  | 1.442 |  |  | 3 | -48.695 | 103.7 | 22.0 | 0.000 |
| THERMAL | 40.490 |  | -0.025 |  |  | -1.605 | 3 | -49.981 | 106.3 | 24.6 | 0.000 |
| DTR | 46.030 |  |  |  |  | -1.847 | 2 | -52.377 | 108.9 | 27.2 | 0.000 |
| D2B | -1.732 |  | -0.031 |  |  |  | 2 | -54.264 | 112.7 | 31.0 | 0.000 |
| NULL | -2.838 |  |  |  |  |  | 1 | -58.519 | 119.1 | 37.4 | 0.000 |
| CP | -3.690 | 0.065 |  |  |  |  | 2 | -58.465 | 121.1 | 39.4 | 0.000 |

Table S23. Full AICc table for patch-scale pellets – Camas – Summer

| Model | (Int) | CP | D2B | MT | AC | DTR | df | logLik | AICc | delta | weights |
| --- | --- | --- | --- | --- | --- | --- | --- | --- | --- | --- | --- |
| D2B | -3.427 |  | -0.025 |  |  |  | 2 | -22.699 | 49.6 | 0.0 | 0.179 |
| FOOD+THERMAL | 32.910 | 0.457 | -0.044 | -1.663 |  | -1.106 | 5 | -19.284 | 49.6 | 0.0 | 0.179 |
| FOOD+SAFETY | -9.329 | 0.574 | -0.046 | -1.582 | 0.841 |  | 5 | -19.556 | 50.1 | 0.5 | 0.139 |
| NULL | -4.609 |  |  |  |  |  | 1 | -24.545 | 51.2 | 1.6 | 0.080 |
| GLOBAL | 132.300 | 0.391 | -0.042 | -1.626 | -4.010 | -3.732 | 6 | -18.916 | 51.3 | 1.7 | 0.076 |
| THERMAL | 8.792 |  | -0.022 |  |  | -0.336 | 3 | -22.627 | 51.6 | 2.0 | 0.066 |
| SAFETY | -3.324 |  | -0.025 |  | -0.131 |  | 3 | -22.694 | 51.8 | 2.2 | 0.060 |
| DTR | 24.980 |  |  |  |  | -0.807 | 2 | -24.002 | 52.2 | 2.6 | 0.049 |
| SAFETY+THERMAL | 115.700 |  | -0.022 |  | -4.764 | -3.172 | 4 | -21.882 | 52.4 | 2.8 | 0.044 |
| CP | -6.765 | 0.198 |  |  |  |  | 2 | -24.325 | 52.8 | 3.2 | 0.036 |
| AC | -5.058 |  |  |  | 0.736 |  | 2 | -24.364 | 52.9 | 3.3 | 0.034 |
| MT | -4.623 |  |  | -0.214 |  |  | 2 | -24.382 | 53.0 | 3.4 | 0.033 |
| FOOD | -9.606 | 0.457 |  | -0.574 |  |  | 3 | -23.601 | 53.6 | 4.0 | 0.024 |

Table S24. Full AICc table for patch-scale bites – Cedar Gulch – Summer

| Model | (Int) | CP | D2B | MT | AC | DTR | df | logLik | AICc | delta | weights |
| --- | --- | --- | --- | --- | --- | --- | --- | --- | --- | --- | --- |
| AC | -2.989 |  |  |  | 4.460 |  | 2 | -46.023 | 96.2 | 0 | 0.4454 |
| SAFETY+THERMAL | -68.320 |  | -0.013 |  | 5.294 | 1.832 | 4 | -44.361 | 97.3 | 1.1 | 0.2570 |
| SAFETY | -2.523 |  | -0.011 |  | 4.377 |  | 3 | -45.559 | 97.4 | 1.2 | 0.2444 |
| GLOBAL | -68.500 | -0.010 | -0.013 | -0.063 | 5.321 | 1.841 | 6 | -44.333 | 101.8 | 5.6 | 0.0271 |
| FOOD+SAFETY | -2.721 | 0.015 | -0.012 | -0.058 | 4.375 |  | 5 | -45.533 | 101.9 | 5.7 | 0.0258 |
| NULL | -1.618 |  |  |  |  |  | 1 | -55.666 | 113.4 | 17.2 | 0.0001 |
| D2B | -1.055 |  | -0.015 |  |  |  | 2 | -54.629 | 113.4 | 17.2 | 0.0001 |
| DTR | 30.430 |  |  |  |  | -0.896 | 2 | -55.083 | 114.3 | 18.1 | 0.0001 |
| THERMAL | 26.850 |  | -0.014 |  |  | -0.782 | 3 | -54.203 | 114.7 | 18.5 | 0.0000 |
| CP | -2.733 | 0.082 |  |  |  |  | 2 | -55.479 | 115.1 | 18.9 | 0.0000 |
| MT | -1.618 |  |  | 0.007 |  |  | 2 | -55.665 | 115.5 | 19.3 | 0.0000 |
| FOOD | -2.733 | 0.083 |  | -0.002 |  |  | 3 | -55.479 | 117.3 | 21.1 | 0.0000 |
| FOOD+THERMAL | 25.420 | 0.092 | -0.015 | -0.026 |  | -0.775 | 5 | -53.970 | 118.8 | 22.6 | 0.0000 |
